# The Influence of Tropical Climates on European Constitutions

**Published:** 1846-08

**Authors:** 


ARTICLE VIII.

*The Influence of Tropical Climates on European Constitutions.* By JAMES JOHNSON, M.D., Physician to the late King, etc. and JAMES RANALD MARTIN, Esq., late Presidency Surgeon, and Surgeon to the Native Hospital, Calcutta. From the sixth London edition, with notes by an American Physician. New York: Samuel S. & William Wood, 261 Pearl Street. 1846. 8vo. pp. 624. (From the Publishers.)

*Five Dissertations on Fever.* By GEORGE FORDYCE, M. D., F. R. S., Fellow of the Royal College of Physicians, Senior Physician to St. Thomas' Hospital, and Reader on the Practice of Physic in London. Second American edition, with an introduction. Philadelphia: Ed. Barrington & Geo. D. Haskell. 1846. 8vo. pp. 403. (From the Publishers.)

To the merits of the former of these works it is almost unnecessary to refer, having been so long and so favorably known to the profession, as to have become part of our Classic Medical Literature; and the publishers are entitled to thanks for increasing our facilities for obtaining it. In regard to Dr. Johnson as an author, the writer of his obituary in the Medico-Chirurgical Review, says, "he was remarkable for a facility of composition, a felicitous, though not always a correct style, and an original vigor and raciness of observation and expression, that redeem some faults, and make his works eminently readable. He may be almost called the Cobbett of Medical Literature, the same boldness, terseness, and straight-forwardness being characteristic of the writings of both."

It is this vigor of expression, the terseness and straight-forwardness, which, added to their intrinsic value have given his writings so widely spread a popularity.

Dr. Fordyce, although not an elegant writer, expresses his thoughts clearly and forcibly.

It will be found replete with information of the most practical character, and his treatment is detailed with a profitable minuteness, the author never generalizing on this point. It also has the great merit of being the production of one whom, in addition to his vast learning, was not biased by theories but drew his descriptions from nature, without disguising her to suit a favorite theory. The Editor, Dr. John Bell of Philadelphia, (this being the April No. of the Select Medical Library,) has added an introduction and divided the dissertations into chapters, preceded by copious contents, which, with his index, has materially increased its convenience as a book of reference. . H. S. H.

